# Comparison on the Efficacy between Partially Covered Self-Expandable Metal Stent with Funnel-Shaped Enlarged Head versus Uncovered Self-Expandable Metal Stent for Palliation of Gastric Outlet Obstruction

**DOI:** 10.1155/2018/4540138

**Published:** 2018-04-23

**Authors:** Jung Wan Choe, Jong Jin Hyun, Dong-won Lee, Sang Jun Suh, Seung Young Kim, Sung Woo Jung, Young Kul Jung, Ja Seol Koo, Hyung Joon Yim, Sang Woo Lee

**Affiliations:** Department of Internal Medicine, Korea University Ansan Hospital, Ansan, Republic of Korea

## Abstract

**Background:**

Shape modification has been one of the methods adopted to improve stent patency but has not always translated into positive outcome. The aim of this study was to compare the efficacy of shape-modified partially covered self-expandable metal stent (SEMS) that has enlarged head versus uncovered SEMS for palliation of gastric outlet obstruction (GOO).

**Methods:**

A total of 48 patients underwent insertion of either enlarged-head SEMS (*n* = 24) or uncovered SEMS (uSEMS) (*n* = 24) for palliation of GOO from July 2009 to July 2016. Patients with inoperable or advanced malignancy were included. Technical feasibility and clinical outcomes were compared.

**Results:**

Technical success rate was 100% (24/24) and 95.8% (23/24) for enlarged-head SEMS group and uSEMS group, respectively. Clinical success rate was 87.5% (21/24) and 87.0% (20/23) for enlarged-head SEMS group and uSEMS group, respectively. The gastric outlet obstruction scoring system score significantly improved in both groups (*p* < 0.001 for both). Mean survival was similar between the groups: enlarged-head SEMS group, 99.3 days (range, 19–358 days) versus uSEMS group, 82.1 days (range, 11–231 days) (*p* = 0.418). The mean stent patency also showed no difference between the groups: enlarged-head SEMS group, 87.1 days (range, 8–358 days) versus uSEMS group, 60.4 days (range, 2–231 days) (*p* = 0.204). With enlarged-head SEMS, distal migration did not occur, but proximal migration was observed in four cases.

**Conclusions:**

Distal migration was prevented by shaping the SEMS to have an enlarged head, but improvement in stent patency could not be observed.

## 1. Introduction

Gastric outlet obstruction (GOO) is a complication that can occur in gastric, duodenal, pancreatobiliary, and other malignancies. Placement of self-expandable metal stent (SEMS) has emerged as a good alternative to a bypass operation in relieving GOO symptoms for palliation in patients with inoperable and advanced malignancy or those refusing surgery. According to a systemic review, the overall clinical success rate of SEMS for GOO palliation is high, ranging from 84% to 93%, with a technical success rate ranging from 93% to 97% [1–3]. The less invasive endoscopic treatment yields lower morbidity and mortality, shorter hospital stay, and earlier symptom relief compared to surgery [4]. SEMS can be largely divided into covered SEMS and uncovered SEMS (uSEMS). Many studies have focused on which of the two SEMS showed better patency [5, 6]. Covered SEMS has risk of stent migration, whereas uSEMS is associated with increased chance of restenosis due to tumor ingrowth. Despite different limitations of each stent type, they showed similar patency rates [7]. As a result, there have been various trials to test different methodologies for decreasing migration in covered SEMS, including modifying the design of the stent, increasing the diameter of the stent, or using endoscopic clips to fix the stent in the desired location [8–10]. If the proximal portion of the SEMS is enlarged so as to take on a funnel shape and covering material is applied at the midportion that is in contact with the tumor, both the migration and tumor ingrowth could be expected to be minimized. This study aimed at evaluating the technical and clinical efficacies of partially covered SEMS with enlarged head compared to uSEMS for palliation of GOO.

## 2. Materials and Methods

### 2.1. Patients

A total of 48 patients with malignant GOO underwent endoscopic SEMS placement at Korea University Ansan Hospital from July 2009 to July 2016. Inclusion criteria were as follows: (1) inoperable malignancy or refusal to undergo surgery, (2) absence of additional stricture in the small bowel and colon, and (3) capability to undergo upper endoscopy. Baseline characteristics including demographics, cancer type and stage, site of GOO, general condition (BMI and ECOG performance status), and severity of obstruction presented by the GOO scoring system (GOOSS) were identified. The GOOSS score is an objective evaluation of the efficacy and patency of SEMS in clinical practice [11]. Data were collected by retrospectively reviewing the electronic medical records of patients. This study was approved by the internal review board committee of Korea University Ansan Hospital (AS16173).

### 2.2. Stent Placement

**Figure 1:**
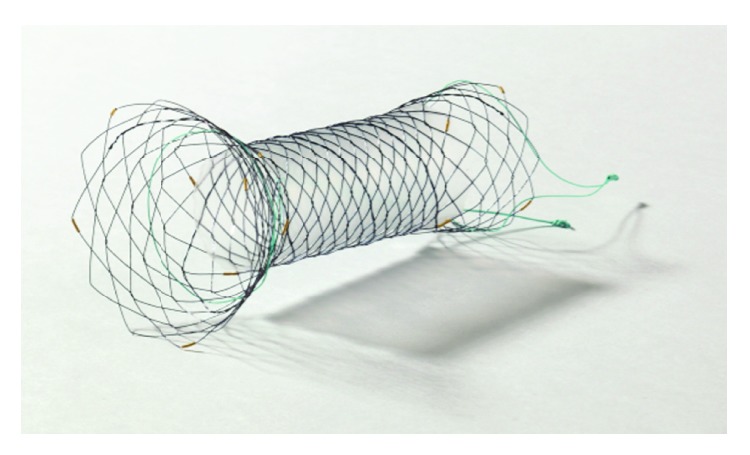
The proximal portion of this partially covered stent takes on a funnel shape in order to prevent distal migration. The midportion is covered with a membrane to prevent tumor ingrowth, whereas both the proximal portion and distal end are uncovered.

SEMSs were inserted using the standard through-the-scope placement technique with either GIF 2T-240 (Olympus Optical, Tokyo, Japan) or duodenoscope (TJF-240 or TJF-260V; Olympus Optical, Tokyo, Japan). From July 2009 to July 2012, uSEMSs were primarily used, and from August 2012 to July 2016, partially covered SEMS with enlarged head was used in the majority cases after its introduction. The length and degree of obstruction were assessed by injecting contrast agent through the stricture and by using a hydrophilic guide wire with radiopaque centimeter markers that was passed through the stricture. When the guide wire was correctly positioned distal to the stricture, the delivery device loaded with the stent was advanced over the guidewire. A stent that was at least a few centimeters longer than the stricture on both sides was chosen to guarantee a disease-free margin and to extend well around curves. Finally, the stent was deployed under continuous fluoroscopic control. Two types of duodenal SEMSs, that is, partially covered SEMS with enlarged head and uSEMS, were used. The partially covered SEMS with enlarged head used in this study (HANAROSTENT® Pylorus/Duodenum Kim's Flare, M.I. Tech, Seoul, Korea) is preloaded into a 10.2 Fr (OD 3.4 mm) delivery sheath. When deployed, it has a funnel-shaped enlarged head in the proximal end intended to prevent distal migration by being fitted at the pylorus or proximal end of the duodenal stricture (Figure 1). The midportion of this stent is covered with a membrane to prevent tumor ingrowth. Both the proximal end (2 cm) and distal end (0.5 ~ 1 cm) of the stent are uncovered. The funnel-shaped enlarged head at the proximal end is 40 mm in diameter that tapers to 20 mm over the length of 2 cm. As for the uSEMSs, the following SEMSs with delivery sheath diameter of 10 Fr (OD 3.33 mm) were used: BONASTENT M-duodenal stent (Standard Sci-Tech, Seoul, Korea), Niti-S D-type pyloric/duodenal stent (Taewoong Medical, Seoul, Korea), and Wallflex duodenal stents (Boston Scientific Corp., Natick, MA). All endoscopic procedures were performed by experienced endoscopists (JJH, SWJ, and SYK) with patients consciously sedated with midazolam (0.05 mg/kg body weight; 1 mg if age > 70 or ASA class III-IV) and meperidine (50 mg; 25 mg if age > 70).

### 2.3. Outcome Measures and Definitions

The primary end points were technical and clinical success. Technical success was defined as successful stent placement at the site of the stricture. Clinical success was defined as relief of GOO symptom or improvement of the GOOSS score 1 week after stent insertion. The secondary end points were duration of stent patency (from the time of stent insertion to the time of stent failure or death) and early (within 1 week) or late (after 1 week) intervention-related complications. Stent failure was defined as stent migration, restenosis due to tumor ingrowth/overgrowth, or any other conditions that caused GOO. Whenever GOO was suspected, CT and upper endoscopy were performed. Patients who had not experienced recurrent obstructive symptoms owing to stent dysfunction were censored at the date of the last follow-up or upon death.

### 2.4. Statistical Analysis

Statistical analyses were performed using IBM SPSS Statistics version 20.0 (IBM, Armonk, NY, USA). Data are expressed as means ± standard deviation (SD) or *n* (%). Categorical variables were compared using the *χ*^2^ tests or Fisher's exact tests, and continuous variables were compared using the independent two-tailed *t*-tests. Analyses of pooled data using univariate and multivariate logistic regression models were conducted to define the independent predictive factors for stent patency. Cumulative stent patency and patient survival were analyzed using the Kaplan–Meier method and were compared by using the log-rank test. Two-sided *p* values < 0.05 were considered to indicate statistical significance.

## 3. Results

### 3.1. Patient Characteristics

A total of 48 patients with GOO caused by malignant tumors who underwent endoscopic SEMS placement at Korea University Ansan Hospital between July 2009 and July 2016 were included. Partially covered SEMS with enlarged head was inserted in 24 patients (enlarged-head group) and uSEMS was inserted in the remaining 24 patients (uSEMS group). The demographic and clinical characteristics of the patients are summarized in Table 1. There were no statistically significant differences between the two groups with regard to age, sex, tumor characteristics, site of obstruction, and length of stenosis, albeit the length of stenosis tended to be longer in the uSEMS group (2.91 ± 1.12 cm versus 2.38 ± 0.71 cm, *p* = 0.06). General conditions represented by BMI and ECOG performance status were also similar between the two groups. The GOOSS scores between the two groups showed no significant differences at baseline before stent placement.

### 3.2. Clinical Outcomes

**Figure 2:**
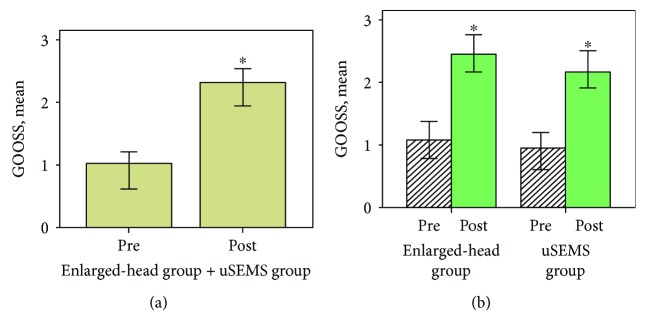
Comparison of mean GOOSS score before and after stent insertion. (a) GOOSS score significantly improved in 47 patients in whom the stent has been successfully placed. (b) When the result is analyzed according to the group, both the enlarged-head group (*n* = 24) and uSEMS group (*n* = 23) showed significantly higher GOOSS score after stent placement. GOOSS: gastric outlet obstruction scoring system; uSEMS: uncovered self-expandable metallic stent. ^∗^*p* value < 0.001.

The overall technical success rate was 97.9% (47/48): 100% (24/24) in the enlarged-head group and 95.8% (23/24) in the uSEMS group. The reason for unsuccessful stent placement in 1 patient from the uSEMS group was difficulty in approaching the stricture segment and passing the guidewire. Among patients who achieved technical success, the clinical success rates of partially covered SEMS with enlarged-head placement and uSEMS placement were 87.5% (21/24) and 87.0% (20/23), respectively (*p* = 1.00). Among 6 patients that failed to achieve clinical success (i.e., improvement in the GOOSS score), 1 patient underwent gastrojejunostomy because the inserted uSEMS did not adequately expand. There were no differences in primary outcomes. The median procedure time, length of stent, chemotherapy after stent placement, oral intake status after stent placement, and median GOOSS score before and after stent placement were not different between the two groups (Table 2). However, when the mean score before stenting was compared with the score after stent placement, improvement was seen for all types of SEMS (Figure 2(a)) in both groups (Figure 2(b)).

### 3.3. Overall Stent Patency and Survival

**Figure 3:**
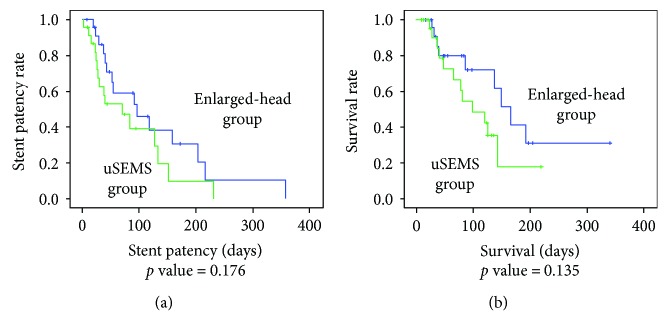
Cumulative stent patency and survival rate. Both the (a) stent patency rate and (b) survival rate seem to be slightly better in the enlarged-head group; there was no statistical significance. uSEMS: uncovered self-expandable metallic stent.

The median stent patency time was 87.1 days for the enlarged-head group compared to 60.4 days for uSEMS group. Although the stent patency tended to be longer for the enlarged-head group, it was not statistically significant (*p* = 0.204). The median survival duration was 99.3 days for the enlarge-head group compared with 82.1 days for the uSEMS group, again without statistical significance (*p* = 0.418) (Table 2). The cumulative stent patency and patient survival were analyzed using the Kaplan–Meier method. The stent patency rate and survival rate of enlarged-head group seemed to be slightly superior to those of uSEMS group, but there were no statistically significant differences (Figure 3).

### 3.4. Complications

**Figure 4:**
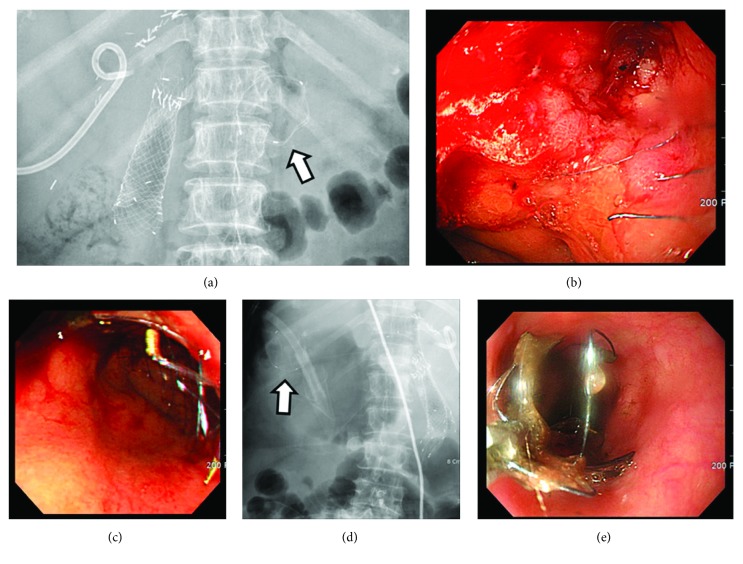
Fracturing of the funnel portion of partially covered SEMS with enlarged head. Detached part of the fractured stent (arrow) is seen on plain abdominal X-ray (a). Fractured proximal end of the stent is observed along with prominent tumor ingrowth (b). The endoscopic view (c) and fluoroscopic view (d) after 2nd partially covered SEMS with enlarged-head placement show that it has been properly deployed over the 1st SEMS. The ring-shaped detached part of the fractured SEMS (arrow) (d) is being removed by rat-tooth forceps (e).

No acute complications, including perforations or aspiration pneumonia, were noted in patients after stent insertion, except for one patient with an inadequate expansion of the uSEMS. There were no stent insertion-related deaths. As for the late complications in the enlarged-head group, fracturing of the funnel (Figure 4) occurred in 2 cases, and tumor ingrowth through the uncovered portion of the SEMS, that is, funnel, occurred in 2 cases. In the uSEMS group, tumor ingrowth occurred in 8 patients. Distal migration of SEMS did not occur in the enlarged-head group, but proximal migration was observed in 4 cases. Proximal migration was observed in 1 patient from the uSEMS group during chemotherapy after stent insertion. All proximally migrated SEMSs fully migrated into the stomach and were endoscopically removed by rat-tooth forceps.

### 3.5. Predictive Factors for Stent Patency

Using univariate and multivariate logistic regression analysis, all possible factors considered to have influence on the stent patency were analyzed. In the univariate analysis, chemotherapy after stent insertion was identified as an independent predictor of stent patency. However, in the multiple regression analysis, none of the variables proved to be significant (Table 3).

## 4. Discussion

In this retrospective study, we showed the efficacy of the partially covered SEMS with enlarged head compared to uSEMS for palliation of GOO. The clinical and technical success rates with this modified SEMS in the management of GOO were 87.5% and 100%, respectively, which were in accordance with the SEMS success rate mentioned in the literatures [7]. Although distal migration was prevented by shaping the partially covered SEMS to have an enlarged head and tumor ingrowth occurred less frequently compared to uSEMS, there was no difference in the stent patency between the two types of SEMS.

Many studies have been focused on determining which type of SEMS showed better patency, covered SEMS or uSEMS [5–7]. Stent migration rarely occurs with uSEMS, but tumor ingrowth is a problem. Tumor ingrowth can be minimized with covered SEMS, but migration, particularly distal migration due to peristalsis, poses a problem. Perhaps due to the advantages and disadvantages of each stent, one type of SEMS had not been shown to be superior over the other with systematic review demonstrating similar patency rates [7]. Until now, much efforts had been put into to overcome the drawbacks of each type of SEMS as follows. Regarding the uSEMS, three methods have been applied to enhance the stent patency. First, uSEMS had been combined with chemotherapy, which would retain the mechanical advantages of the SEMS and the chemical advantages of chemotherapy [12, 13]. In our study, poststent chemotherapy showed a possible protective effect against stent dysfunction. This result is comparable to results of other studies [12–14]. The challenge to this approach is that systemic chemotherapy cannot be uniformly applied, considering that the subjects are in relatively immune-compromised state and have poor performance status due to existing malignancy. Moreover, large prospective series found a significant association between the use of chemotherapy and increased incidence in stent migration [13]. Nevertheless, it would be worth mentioning that there is a risk of bias when performing analysis in this kind of situation where competing risks are present as clearly pointed out by Hamada et al. [15]. Second, simultaneous double placement of a covered SEMS and a uSEMS during the same session had been attempted to decrease both the migration and obstruction [16, 17]. However, the problems of longer procedure time, higher costs, and a lower-than-expected efficacy remain to be solved. Furthermore, a double-layered SEMS, consisting of an outer uncovered stent designed to reduce migration and an inner covered stent to suppress tumor ingrowth, is already on the market [12]. With this SEMS, procedure time can be saved by not having to go through the procedure twice, and the cost is similar to other duodenal SEMS. However, the currently available data shows no definite advantage of double-layered SEMS over a covered SEMS with regard to patency rate. As for covered SEMS, there have been several efforts to reduce the tendency for migration by modifying the design of the stent or mechanical fixation. As a part of this effort, the central portion of a covered SEMS was given a bumpy and wavy external appearance to provide mechanical resistance [9]. However, the migration rate of this new SEMS was still higher than that of uSEMS, albeit not statistically significant [9]. In another attempt to overcome stent migration, SEMS was anchored using endoscopic clips that grasped an adequate amount of the adjacent healthy tissue with one of the wires of the metal stent at its proximal end [8]. However, this technique did not show the desired control over stent migration. Another SEMS technique employed by van den Berg et al. was making a partially covered SEMS with a big cup [10]. This study was prematurely terminated because migration occurred in 50% (3/6) of cases at a relatively early stage. In our study however, the rate of proximal migration was 16.7% (4/24), which would be much lower compared to the study by van den Berg et al. Although the efficacy of the SEMS with a big cup could not be fully evaluated due to the small sample size in their study, high rate of proximal stent migration could clearly be perceived as an obstacle that had to be overcome with this type of design modification. Recently, Shi et al. designed a “tailored” partially covered SEMS and compared it with conventional uSEMS [18]. Two shapes of “tailored” SEMS were used in this study; the proximal end of one stent was cup shaped (53.3 ± 5.5 mm in diameter and 15 to 20 mm in length) and the other was funnel shaped (33.6 ± 3.6 mm in diameter and 25 to 30 mm in length). These “tailored” partially covered SEMS reduced tumor ingrowth/outgrowth but did not prevent migration of stent or increase survival. The overall outcomes of their study were similar with those of our study. However, whereas the “tailored” partially covered SEMS had to be placed fluoroscopically since it needed larger than 6 mm delivery system, all the partially covered SEMSs with enlarged head in the current study were placed using through-the-scope method under direct endoscopic visualization. Through-the-scope method ensured technical ease and proved to be less time-consuming (mean procedure time, 15.8 min versus 56 min) [19]. Takahara et al. also conducted a study with a partially covered SEMS with a large-bore flare proximal end, a concept similar to that of the present study [20]. Although this SEMS proved to be safe and effective for the palliation of malignant GOO, stent migration could not be overcome with a migration rate of 23%. However, it is noteworthy that all migrations in their study were distal. This is in contrast with the direction of migration observed in our study where proximal and not distal migration was the problem. Therefore, the size (40 mm) and radial force of the funned-shaped enlarged head used in our study seem to be more ideal in preventing distal migration compared to the 25 mm-sized proximal flare with low radial force used in their study.

As expected, tumor ingrowth occurred less frequently in the enlarged-head group and distal migration was prevented. Thus, the partially covered SEMS with enlarged head could be considered a promising stent option for durable palliation of symptomatic GOO if higher proximal migration rate compared to the uSEMS could be overcome. One of the most plausible mechanisms of proximal migration with covered SEMS could be explained by the soap bar effect which was described by Adam et al. [21]. According to his description, peristalsis combined with the conical shape of the stent within a relatively short stricture and the smooth surface of the covered stent that is in contact with the tumor would have resulted in upward forces to push the stent in a proximal direction. Therefore, lowering the axial force would help reduce the proximal migration due to soap bar effect. Another improvement that can be made with the partially covered SEMS with enlarged head in prolonging the stent patency would be to further decrease tumor ingrowth which occurred in 2 patients. The location of the tumor ingrowth was at the funnel portion that was not covered by the covering material. This could have occurred because the part where the uncovered funnel portion meets the covered portion of the SEMS and becomes anchored is where the obstruction by the tumor begins. Therefore, in order to increase stent patency, the SEMS could be modified to have part of the funnel covered with the covering material.

This study has some limitations. First, it was a retrospective study with a small number of cases during a long study period at a single center, albeit a tertiary referral center. Second, several uSEMSs from a different manufacturer were used. Thus, the axial force and the radial force would not have been uniform and may have affected the clinical success, complications, and stent patency in the uncovered SEMS group. However, since no significant differences in terms of outcomes among the manufacturers of the uSEMS have been demonstrated in a systematic review, the influence of having used SEMS from various manufacturers could have been minimal [22]. Third, lack of specific information on the impact of possible confounding factors for stent patency, such as chemotherapy response and histologic differentiation of tumors, could not be analyzed for being a retrospective study. Fourth, determining the exact cause of stent dysfunction was not always possible because enrolled patients tended to die of complications from malignancy rather than GOO. Regular endoscopic surveillance or upper GI series study would be helpful in determining the cause but would not be a very realistic approach in terminal cancer patients.

In conclusion, although distal migration was prevented by shaping the SEMS to have an enlarged head, there was no difference in the stent patency between the two types of SEMS. Therefore, choice of SEMS type should be left at the discretion of the physician depending on the characteristics and site of the stricture until further progress is made with SEMS.

## Figures and Tables

**Table 1:** Demographic and clinical characteristics of the 48 included patients.

Characteristics	Enlarged-head group (*n* = 24)	uSEMS group (*n* = 24)	*p* value
Age, years	70.5 ± 10.4	73.0 ± 11.9	0.447
Sex, male	11 (45.8)	12 (50.0)	0.781
Tumor characteristics			0.420
Pancreatic cancer	10 (41.6)	10 (41.7)	
Cholangiocarcinoma	7 (29.2)	3 (12.5)	
Duodenal cancer	1 (4.2)	0 (0.0)	
Gastric cancer	2 (8.3)	7 (29.2)	
Gallbladder cancer	4 (16.7)	3 (12.5)	
Ampulla of Vater cancer	0 (0.0)	1 (4.1)	
Site of obstruction			0.457
Pylorus	5 (20.8)	6 (25.0)	
First part of duodenum	13 (54.2)	8 (33.3)	
Second part of duodenum	5 (20.8)	7 (29.2)	
Third part of duodenum	1 (4.2)	3 (12.5)	
Length of stenosis	2.38 ± 0.71	2.91 ± 1.12	0.06
General condition			
BMI, kg/m^2^	20.54 ± 3.47	20.13 ± 3.44	0.686
ECOG performance status	2 (2–4)	2 (2-3)	0.519
Severity of obstruction			
GOOSS score			0.512
0 no oral intake	5 (20.8)	5 (20.8)	
1 liquids only	12 (50.0)	16 (66.7)	
2 soft solid	7 (29.2)	3 (12.5)	
3 low-residue or normal diet	0 (0.0)	0 (0.0)	

Values are *n* (%) or mean ± SD or median (range). SEMS: self-expandable metallic stent; BMI: body mass index; GOOSS: gastric outlet obstruction scoring system.

**Table 2:** Clinical outcomes of 47 patients who achieved technical success.

	Enlarged-head group (*n* = 24)	uSEMS group (*n* = 23)	*p* value
Clinical success	21/24 (87.5)	20/23 (87.2)	1.00
Short-term outcomes			
Median procedure time (min)	13 (5–40)	14 (5–30)	0.901
Length of stent	9.08 ± 1.50	8.65 ± 2.30	0.46
Chemotherapy after stent placement	3/24 (12.5)	6/23 (26.1)	0.286
Oral intake status after stent placement			0.197
Liquid	3 (12.5)	4 (17.4)	
Soft solid	7 (29.2)	12 (52.2)	
Low-residual or full diet	14 (58.3)	6 (26.1)	
Median GOOSS			
Prestent placement	1 (0–2)	1 (0–2)	0.505
Poststent placement	3 (1–3)	2 (1–3)	0.200
Long-term outcomes			
Recurrent symptoms	9 (37.5)	8 (34.8)	1.00
Stent patency, days	87.1 (8–358)	60.4 (2–231)	0.204
Survival duration, days	99.3 (19–358)	82.1 (11–231)	0.418
Tumor ingrowth	2 (8.3)	8 (34.8)	0.036
Stent migration	4 (16.7)	1 (4.34)	0.348

Values are *n* (%) or mean ± SD or median (range). SEMS: self-expandable metallic stent; GOOSS: gastric outlet obstruction scoring system.

**Table 3:** Univariate and multivariate logistic regression analysis for the stent patency.

Stent patency	OR (95% C.I.)	*p* value
*Univariate analysis*		
Chemotherapy after stent insertion	1.937 (0.131 ~ 2.540)	0.032
Survival	0.722 (0.215 ~ 2.427)	0.599
Clinical success	4.833 (0.779 ~ 30.005)	0.091
Stent type		
Enlarged head	Reference	
Uncovered	1.371 (0.408 ~ 4.614)	0.610
Obstruction site		
Pylorus	Reference	
First part of duodenum	1.429 (0.303 ~ 6.737)	0.229
Second part of duodenum	0.800 (0.149 ~ 4.297)	0.795
Third part of duodenum	1.143 (0.077 ~ 16.947)	0.923
Stricture length	1.138 (0.595 ~ 2.174)	0.696
Stent length	0.761 (0.542 ~ 1.068)	0.114
Cancer etiology		
Cholangiocarcinoma	Reference	
Pancreatic cancer	2.800 (0.562 ~ 13.952)	0.209
Stomach cancer	8.000 (0.711 ~ 90.001)	0.092
*Multivariate analysis*		
Clinical success	4.833 (0.779 ~ 30.005)	0.091

## References

[1] Boskoski I., Tringali A., Familiari P., Mutignani M., Costamagna G. (2010). Self-expandable metallic stents for malignant gastric outlet obstruction. *Advances in Therapy*.

[2] Dormann A., Meisner S., Verin N., Wenk Lang A. (2004). Self-expanding metal stents for gastroduodenal malignancies: systematic review of their clinical effectiveness. *Endoscopy*.

[3] Hamada T., Hakuta R., Takahara N. (2017). Covered versus uncovered metal stents for malignant gastric outlet obstruction: systematic review and meta-analysis. *Digestive Endoscopy*.

[4] Lee S. M., Kang D. H., Kim G. H., Park W. I., Kim H. W., Park J. H. (2007). Self-expanding metallic stents for gastric outlet obstruction resulting from stomach cancer: a preliminary study with a newly designed double-layered pyloric stent. *Gastrointestinal Endoscopy*.

[5] Kim C. G., Choi I. J., Lee J. Y. (2010). Covered versus uncovered self-expandable metallic stents for palliation of malignant pyloric obstruction in gastric cancer patients: a randomized, prospective study. *Gastrointestinal Endoscopy*.

[6] Maetani I., Mizumoto Y., Shigoka H. (2014). Placement of a triple-layered covered versus uncovered metallic stent for palliation of malignant gastric outlet obstruction: a multicenter randomized trial. *Digestive Endoscopy*.

[7] Pan Y. M., Pan J., Guo L. K., Qiu M., Zhang J. J. (2014). Covered versus uncovered self-expandable metallic stents for palliation of malignant gastric outlet obstruction: a systematic review and meta-analysis. *BMC Gastroenterology*.

[8] Kim I. D., Kang D. H., Choi C. W. (2010). Prevention of covered enteral stent migration in patients with malignant gastric outlet obstruction: a pilot study of anchoring with endoscopic clips. *Scandinavian Journal of Gastroenterology*.

[9] Lee H., Min B. H., Lee J. H. (2015). Covered metallic stents with an anti-migration design vs. uncovered stents for the palliation of malignant gastric outlet obstruction: a multicenter, randomized trial. *The American Journal of Gastroenterology*.

[10] van den Berg M., Walter D., Vleggaar F., Siersema P., Fockens P., van Hooft J. (2014). High proximal migration rate of a partially covered “big cup” duodenal stent in patients with malignant gastric outlet obstruction. *Endoscopy*.

[11] Adler D. G., Baron T. H. (2002). Endoscopic palliation of malignant gastric outlet obstruction using self-expanding metal stents: experience in 36 patients. *The American Journal of Gastroenterology*.

[12] Park C. I., Kim J. H., Lee Y. C. (2013). What is the ideal stent as initial intervention for malignant gastric outlet obstruction?. *Digestive and Liver Disease*.

[13] Kim J. H., Song H. Y., Shin J. H. (2007). Metallic stent placement in the palliative treatment of malignant gastroduodenal obstructions: prospective evaluation of results and factors influencing outcome in 213 patients. *Gastrointestinal Endoscopy*.

[14] Kim C. G., Park S. R., Choi I. J. (2012). Effect of chemotherapy on the outcome of self-expandable metallic stents in gastric cancer patients with malignant outlet obstruction. *Endoscopy*.

[15] Hamada T., Nakai Y., Isayama H., Sasaki T., Koike K. (2013). Competing risk: a potential risk factor for misleading results of length of stent patency. *Endoscopy*.

[16] Song G. A., Kang D. H., Kim T. O. (2007). Endoscopic stenting in patients with recurrent malignant obstruction after gastric surgery: uncovered versus simultaneously deployed uncovered and covered (double) self-expandable metal stents. *Gastrointestinal Endoscopy*.

[17] Jung G.-S., Song H.-Y., Seo T.-S. (2002). Malignant gastric outlet obstructions: treatment by means of coaxial placement of uncovered and covered expandable nitinol stents. *Journal of Vascular and Interventional Radiology*.

[18] Shi D., Ji F., Bao Y.-s., Liu Y.-p. (2014). A multicenter randomized controlled trial of malignant gastric outlet obstruction: tailored partially covered stents (placed fluoroscopically) versus standard uncovered stents (placed endoscopically). *Gastroenterology Research and Practice*.

[19] Shi D., Bao Y.-s., Liu Y.-p. (2013). Individualization of metal stents for management of gastric outlet obstruction caused by distal stomach cancer: a prospective study. *Gastrointestinal Endoscopy*.

[20] Takahara N., Isayama H., Nakai Y. (2017). A novel partially covered self-expandable metallic stent with proximal flare in patients with malignant gastric outlet obstruction. *Gut and Liver*.

[21] Adam A., Morgan R., Ellul J., Mason R. C. (1998). A new design of the esophageal Wallstent endoprosthesis resistant to distal migration. *American Journal of Roentgenology*.

[22] Hori Y., Naitoh I., Hayashi K. (2017). Predictors of outcomes in patients undergoing covered and uncovered self-expandable metal stent placement for malignant gastric outlet obstruction: a multicenter study. *Gastrointestinal Endoscopy*.

